# Optimized Transferosomal Bovine Lactoferrin (BLF) as a Promising Novel Non-Invasive Topical Treatment for Genital Warts Caused by Human Papiluma Virus (HPV)

**Published:** 2018

**Authors:** Naghmeh Hadidi, Mostafa Saffari, Mehrdad Faizi

**Affiliations:** a *Department of Clinical Research and EM Microscope, Pasteur Institute of Iran (PII), Tehran, Iran.*; b *Department of Medical Nanotechnology and Pharmaceutics, School of Pharmacy, Islamic Azad University of Medical Sciences, Tehran, Iran.*; c *Department of Pharmacology and Toxicology, School of Pharmacy, Shahid Beheshti University of Medical Sciences (SBMU), Tehran, Iran.*

**Keywords:** Transferosomal lactoferrin, Genital warts, HPV, BLF, MTT assay

## Abstract

Human papillomavirus (HPV) cause common warts, laryngeal papilloma, and genital condylomata and might lead to development of cervical cancer. Lactoferrin (LF) is a member of the transferrin family, which has antiviral activity against HPV-16.  LF is an important player in the defense against pathogenic microorganisms and has also been shown to have activity against several viruses including herpesvirus, adenovirus, rotavirus, and poliovirus. Bovine LF (BLF) has been reported to be a more potent inhibitor of HPV entry in comparison to human LF. The goal of the present study is to formulate, evaluate and optimize transfersomal vesicles as a non-invasive transdermal delivery system which assumed to be a suitable for treatment of genital warts. Transfersomes have been prepared by two methods including reverse phase evaporation and thin film hydration with different ratios of cholesterol: lecithin: DOTAP in the presence of SDS or Tween 80. The transferosomes were then evaluated regarding size, polydispersity, and LF loading. *In-vitro* release studies in pH 5.3 and 7.4, stability evaluation in 4 °C and 25 °C, and TEM imaging hve been performed on optimized transferosomal lactoferrin. The optimized transferosomes were found to have 100 nm sizes with good polydispersity index and encapsulation efficiency of 91% for lactoferrin as well as sustained release of lactoferrin during 24 h. Transferosomal lactoferrin efficacy was evaluated by MTT assay. It was seen that the viral inhibitory concentration (IC50) of transfersomal lactoferrin has been significantly improved to nearly one tenth in comparison to free lactoferrin.

## Introduction

Human papillomavirus (HPV) is a naked DNA virus belonging to the family of Papillomaviridae. It infects basal cells in mucosa or skin. Also, it is strongly dependent on the differential status of the epithelium for its viral life cycle. This would result in the development of cervical cancer, condyloma, and other infections of the genital and respiratory tracts (1,2). It has been estimated that global HPV prevalence is 11.7%. Sub-Saharan Africa (24.0%), Eastern Europe (21.4%), and Latin America (16.1%) showed the highest prevalence in HPV related infectious diseases. Age-specific HPV distribution indicates that in Africa and America, the first peak is at younger ages (<25 years) and again rebound at older ages (⩾ 45 years). HPV-16, HPV-18, HPV-52, HPV-31, and HPV-58 are the 5 most common HPV genotypes worldwide (3, 4).

Lactoferrin (LF), a multifunctional iron binding glycoprotein, is present in almost all human biological fluids and plays an important role in the innate immune system. It is a low-cost protein derived from milk and displays no toxic side effects. LF possesses various biological functions such as immunomodulatory, antiviral, antibacterial, antitumor, antifungal, and anti-inflammatory (5). There is evidence that LF is capable of binding the LPS binding protein and the CD14 receptor during cell immunity process. The major challenge facing LF is its poor *in-vivo* stability. It also undergoes rapid degradation by proteolytic enzymes and this would hinder its wide antimicrobial and antiviral application (7 -8). Lactoferrin’s iron capturing ability will lead to inhibition of microbial growth, motility modulation, aggregation and biofilm formation of pathogenic bacteria. On the other hand, lactoferrin interacts with viral surfaces by inhibiting virus adhesion and entrance into host cells and this would lead to its antiviral activity (7, 8). 

LF and transferrin are similar in many aspects but they possess different functions *in-vivo*. Transferrin seems to interfere with iron uptake by cells, whereas LF exerts a protective function by making brick in the mucosal wall. LF cationic nature and its capability of retaining iron at acidic pH would be helpful in infection and inflammation sites, binding to negatively charged viral and microbial surfaces as well as DNA, heparin, and glycosaminoglycans (8). The protective LF antiviral and antimicrobial effect has been widely demonstrated in a large number of *in-vitro* and *in-vivo *studies (1-9). Different mechanisms have been assumed for LF in preventing viral infection (6). It includes binding to viral particles (A), the binding to heparan sulfate glycosaminoglycans (HSGA) (B), the binding to viral receptors (C), and intracellular localization (D), involving apoptosis or inflammatory pathways with a dose-dependent relationship (1, 2, 8, 9). It has be stated that bovine LF (bLF) was a more potent inhibitor of HPV entry than human LF (hLF) (1, 8-9).

Accessibility and ease of application have made skin an ideal route for drug administration. However, skin application is somehow limited because of the barrier nature of stratum corneum. Low penetration capacity would affect range of molecules that can achieve therapeutic amounts following skin application. Transfersomes, new class of liposomes, were first described by Cevc etal. (10, 11 and 12). They had been categorized as deformable, highly deformable, elastic or ultra-flexible vesicles. Transfersomes are reported to improve *in-vitro *skin delivery (12-15) and *in-vivo* penetration to achieve therapeutic amounts that are comparable with subcutaneous injection (16-17).

Lactoferrin has been informed to have antibacterial and antiviral effects (18-25). So that, the goal of the present study is to formulate, evaluate and optimize transfersomal vesicles as a highly deformable liposome via transdermal drug delivery system for the lactoferrin assumed to be a suitable non-invasive transdermal delivery system for treatment of genital warts caused by HPV.

## Experimental

*Materials.*

The bovine lactoferin BLF 99% purity was obtained from BOC science (USA), soy lecithin E80 and cholesterol was purchased from lipoid (US). Tween 80 and DOTAP were bought from Merck (Germany) sourced from Sigma (Germany). Phosphate Buffer Saline (PBS) was prepared from thermoscientific (US). Sodium deoxycholate (SDC) was obtained from sigma (Germany). Ethanol and all the other chemicals were research grade. All chemicals were used as received without further purification. Deionized (DI) water (specific resistivity higher than 18.2 MΩcm) from Milli-Q plus 185 (Millipore) water purification systems was utilized to make all solutions. UP200Ht, Hielscher, Germany was used as ultrasonic processor. 

The morphology was investigated by Transmission electronic microscope (TEM), Zeiss, EM900, Germany.

*Preparation of Tranferosomal Lactoferin*

*Reverse phase evaporation method*

The required quantities of lipid mixture containing Soya lecithin, cholesterol and DOTAP were added to tween 80 as surfactant. They were then dissolved in ethanol by gentle shaking and being stirred in RT (Room Temperature) for 30 min until a yellow transparent solution obtained. Aqueous Lactoferrin was added to this organic phase at the concentration of 5 mg/mL. The resulted emulsion was further stirred for about 30 min. It was sonicated in a water sonication bath for about 4 min. Then it was transferred into a round bottom flask containing 5 grams of glass pearl and installed in to a rotary evaporator. The thin gel layer was formed by vacuum application on rotary evaporator for 30 min at 25 °C, 600 mm/hg pressure and 90 rpm. The jelly like film was then hydrated with PBS (phosphate buffer slain) and vacuum was applied for another half an hour. Obtained transferosmes were sonicated for 3 cycles of 2 min intermittently with ultrasound sonotrode (Heilscher, Germany) with 90% of amplitude power. The transferosomal lactoferrins were observed under Transmission Electronic Microscope (TEM). They were further evaluated for size and lactoferrin loading. Transferosomal lactoferrin was stored in refrigerator at 4 °C. Composition of transferosomes is given in table 1.

*Thin film hydration method*

Required quantities of lipid mixture containing Soya lecithin, cholesterol, and DOTAP were dissolved in ethanol being stirred in RT (Room Temperature) for 30 min until a yellow transparent solution was obtained. It was transferred into a round bottom flask containing 5 grams of glass pearl and installed in to a rotary evaporator. The thin film was formed by vacuum application on rotary evaporator for 10 min at 25 °C, 600 mm/hg pressure and 90 rpm. The film was kept overnight at room temperature to be completely dried. The thin lipid film was hydrated by aqueous solution of Lactoferrin at the concentration of 5 mg/mL in PBS slowly within 30 min. The resulted emulsion was further treated on a rotary evaporator for another 2 h. Obtained transferosmes were sonicated for 3 cycles of 2 min intermittently with ultrasound sonotrode (Heilscher, Germany) with 90% of amplitude power. The transferosomal lactoferrins were observed under Transmission Electronic Microscope (TEM). They were further evaluated for size and lactoferrin loading. Transferosomal lactoferrin was stored in refrigerator at 4 °C. Composition of transferosomes has been shownin Table 1.

*Characterization of transferosomal lactoferrin*

All transferosomal lactoferrin formulations were prepared using two methods and characterized by Nanosizer (Malvern, Germany) regarding particle size distribution to ensure homogeneity and size uniformity. They were also evaluated for entrapment efficiency (EE %), drug content, and *in-vitro* release studies. 

*Determination of size and morphology by TEM*

Transmission Electron Microscopy (TEM) was used to determine the shape, size, and surface morphology of the transferosomes. One drop of the sample tests was put on a formvar coated grid and was negatively stained with PTA2%. The grids were put aside for 1 h to be completely dried. They were seen by TEM, Zeiss EM900, 80 kV in order to confirm both shape and size. 

*Mean Diameter and zeta potential measurement*

The mean diameter, size distribution, and zeta potential of lactoferrin loaded transferosomes were determined by dynamic light scattering (DLS), using Malvern Zeta Sizer, UK. 

*Measurement of drug content and entrapment efficiency (EE %)*

1 mL of transferosomal lactoferrin was taken and diluted with 9 mL phosphate buffer saline pH = 7.4 It was ultracentrifuged at 140000 rpm for 30 min at -4 °C. The pellet formed after centrifugation was disrupted with 1:1 ratio with ethanol 50%. It was then centrifuged at 14000 rpm for 10 min at -4 °C. 1mL of this solution was taken and suitable dilutions were made and analysed by UV spectrophotometer at 280 nm which gives the concentration of the entrapped drug. The concentration of drug in supernatant and pellet collectively gives the amount of drug present in 1mL of suspension. % drug content and % entrapment efficiency (EE%) were calculated based on equations 1, and 2, respectively.

Equation 1: 

% drug content = practical drug content/theoretical drug content ×100 

Equation 2: 

% entrapment efficiency = Total drug added-unentrapped drug/ Total drug added 

*In-vitro drug diffusion study*

Drug diffusion was measured against cellulose acetate in franz diffusion cell. 1 mL of transferosomal lactoferrin was taken in donor compartment and 25 mL of phosphate buffer saline with pH = 7.4 was taken in receiver compartment at 37 ° C. Aliquots of 5mL of samples were withdrawn at specific times for 24 h. The withdrawn amount was replaced with the buffer to maintain sink conditions. Drug release study was investigated by the amount of drug passed through cellulose acetate membrane into PBS containing receiver compartment. 

The samples were analyzed by UV spectrophotometer at 280 nm. The percentage of drug released was reported based on n = 6 in a time period of 24 h.

*Stability Studies*

Physical stability tests were carried out to investigate the transferosomes aggregation and lactoferrin leakage during storage. Transferosomal lactoferrin was stored in transparent vials sealed with rubber aluminum cap temperature and 4 °C and 25 °C for three months. 

The stability was evaluated by size and EE% measurement over a three-month period. The samples from each vesicle were withdrawn monthly, and they were evaluated as described previously.

*MTT assay*

*In-vitro* efficacy of transfersomal lactoferrin and cell viability on Hela cells was investigated by MTT assay according to ISO 10993-5. Hela cells (5 × 103 cells/well) were cultured in a 96-well plate at 37 °C for 24 hr. After 24 h culture, the medium was replaced with test samples and controls , then after 72 h of treatment, the medium again replace with a fresh medium and the cultures were assessed with MTT in triplicates. The cells were then exposed to various concentrations of BLF (Bovine Lactoferrin) from 0.0625-2.5 mg/mL, T-BLF (Transfersomal Bovine Lactoferin) from 0.0625-1.25 mg/mL, T (empty transfersomes) at 0.626 and 1.25 mg/mL and 5FU with concentration of 1 and 1.5 µg/mL for 72 h. Supernatants were removed from each well and then were washed twice by PBS. Finally, 20 µl of MTT solution (5 mg/ mL) was added to each well. After incubation for another 4 h, the resultant formazan crystals were dissolved in 100 µL isopropanol and the absorbance intensity measured by a microplate reader (Bio-RAD 680, USA) at 545 nm with a reference wavelength of 620 nm. All experiments were performed in quadruplicate, and the relative cell viability (%) was expressed as a percentage relative to the untreated control cells. 

## Results

*Characterization of Transferosomal Lactoferrin*

**Figure 1:**
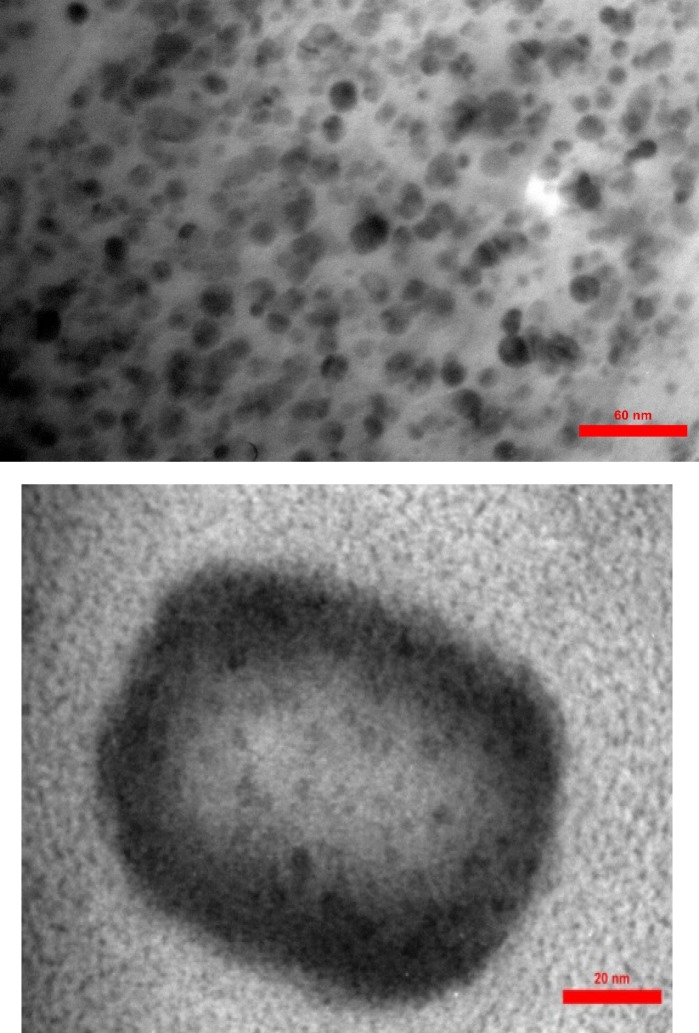
Morphology investigation of transfersomal lactoferrin by TEM

**Figure 2:**
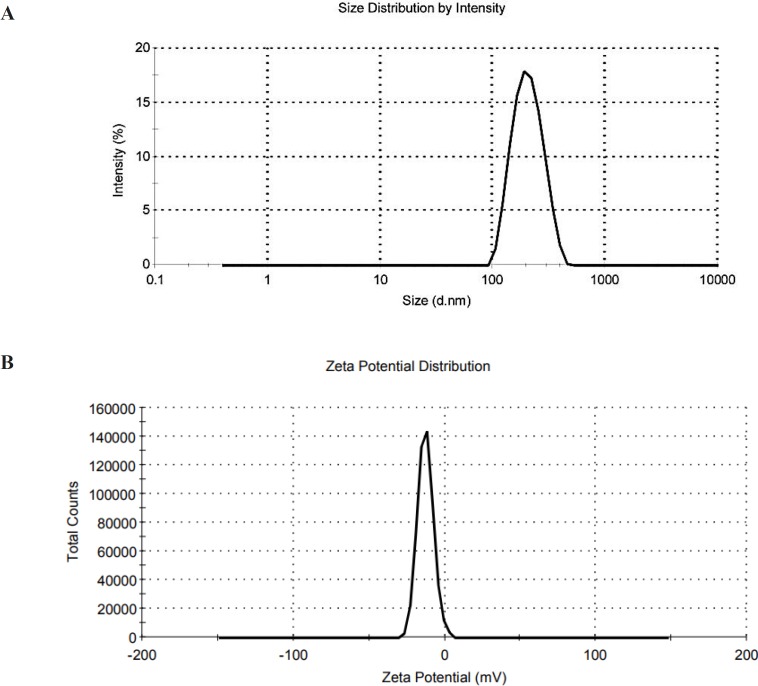
**( **A) Size distribution of O1 formulation, PDI = 0.1, (B) Zeta potential of O1

The prepared transferosomes were semi-spherical in shape (Figure 1). The mean diameter of lactoferrin loaded transferosomes determined by DLS were in the range of 95-237 nm with highest homogeneity reported (PDI = 0.1) for C1, D5 and O1 formulations (Figure 2). The mean encapsulation efficiency of the drug in transfersomes was obtained as 47%, 79%, and 91% for C1, D5, and O1 formulations, respectively (Table 1).

**Table1:** Formulation variables used in preparation of BLF transferosomes

**No**	**Formulation**	**method**	3 **LF concentration (mg/mL)**	**Surfactant**	4 **S: L** **ratio**	**cholesterol**	**DOTAP**	**Size (nm)**	5 **EE%**	**Zeta (mv)**
1	S1	TFH1	5	Tween 80	1:10	No	No	237.0	24	+0.1
2	S2	TFH	5	SDC	1:10	No	No	2666.0	20	+0.5
3	S3	TFH	5	Tween 80	1:10	No	No	160.0	30	+1
4	S4	TFH	5	Tween 80	2:9	No	No	108.0	38	+2.5
5	S5	TFH	5	Tween 80	3:8	No	No	98.0	43	+10
6	S6	2RPE	5	Tween 80	1:10	No	No	99.0	44	-26
7	S7	RPE	5	Tween 80	2:9	No	No	73.5	42	-25
8	S8	RPE	5	Tween 80	3:8	No	No	112	74	-30
9	D1	TFH	5	Tween 80	61:9:1	No	Yes	134	24	+15
10	D2	TFH	5	Tween 80	62:8:1	No	Yes	134	32	+25
11	D3	TFH	5	Tween 80	63:7:1	No	Yes	136	38	+17
12	D4	RPE	5	Tween 80	61:9:1	No	Yes	83	46	+20
13	D5	RPE	5	Tween 80	62:8:1	No	Yes	95	79	+20
14	D6	RPE	5	Tween 80	63:7:1	No	Yes	171	63	+20
15	C1	RPE	5	Tween 80	71:9:1	Yes	No	128	47	-25
16	C2	RPE	5	Tween 80	73:7:1	Yes	No	132	39	-18
17	C3	RPE	5	Tween 80	75:5:1	Yes	No	132	37	-19
18	O1	RPE	5	Tween 80	83:6:2:1	Yes	Yes	128	91	-35

1 TFH: Thin Film Hydration

2 RPE: Reverse Phase Evaporation

3 LF: Lactoferrin

4 S: L ratio: surfactant: Lipid ratio

5 EE: Entrapment efficiency

6 Surfactant: DOTAP: SPC

7 Surfactant: SPC: cholesterol

8 Surfactant: SPC: DOTAP: cholesterol

**Table 2:** Stability of transfersomes stored in 4 °C and 25 °C

**Formulation code**	**Particle size ( nm)**		**Zeta Potential (mV)**		**EE%**	
	**Initial**	**After 3 months**	**Initial**	**After 3 months**	**Initial**	**After 3 months**
**C1**						
4±1°C	128±15.3	135±15.8	-25± 4.1	-27± 4.1	47±4.0	43±2.0
25±1°C	128±15.3	142±20.5	-25± 4.1	-21± 4.1	47±4.0	35±5.0
**D5**						
4±1°C	95±20.7	101±10.5	+20±4.1	+25±3.5	79±5.0	75±1.1
25±1°C	95±20.7	130±15.5	+20±4.1	+19±4.5	79±5.0	68±4.0
**O1**						
4±1°C	128±14.3	132±12.7	+35± 3.5	+33±3.7	91±3.5	88±0.9
25±1°C	128±14.3	140±12.7	+35± 3.5	+37±4.2	91±3.5	83±1.2

**Figure 3:**
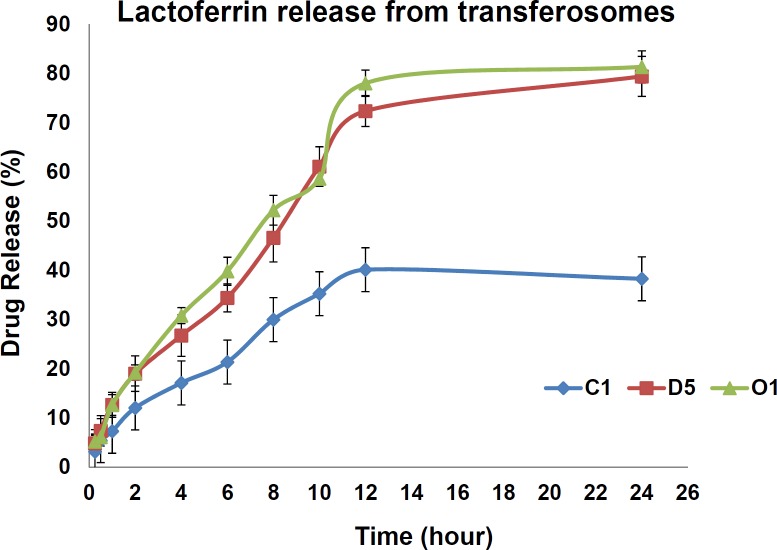
Comparative Cumulative Release (%) of Lactoferrin from 3 optimized formulations, C1, D5 and O1 (n = 3)

**Figure 4:**
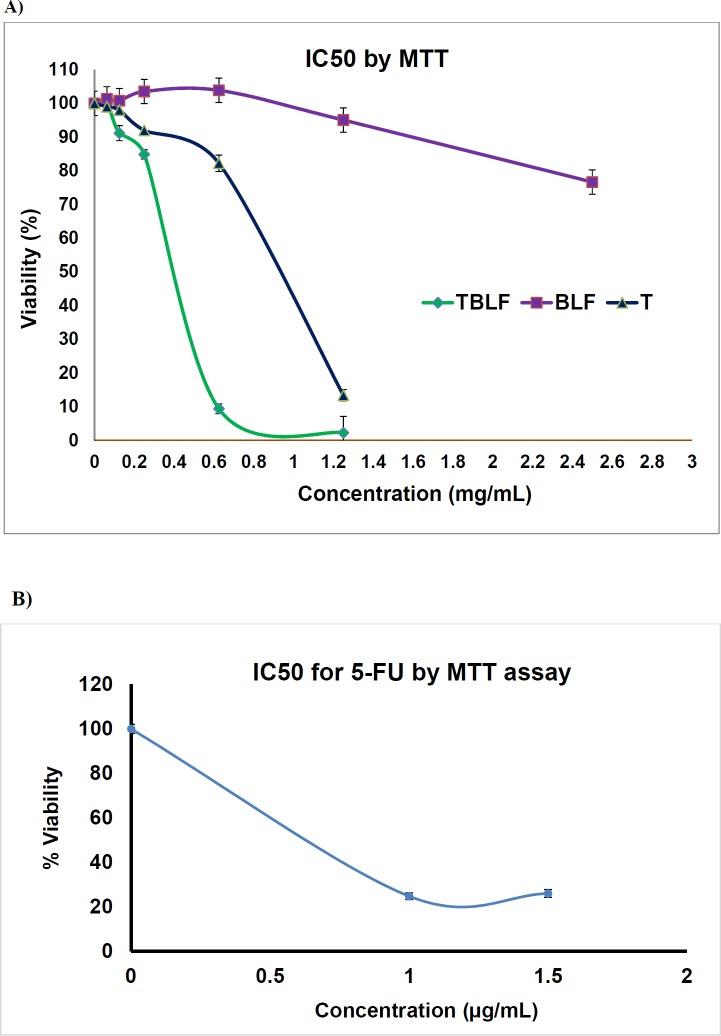
IC_50_ evaluation of Hela cell lines after 72 h exposure to A) TBL (transfersomal Bovine Lactoferrin), 1.25,0.625,0.25,0.125,0.0625 mg/mL , BLF ( Bovine lactoferrin) 2.5,1.25,0.625,0.25,0.125,0.0625 mg/mL, T (empty transfersom) 1.25, 0.625 mg/mL , B) 5-FU 1 and 1.5 µg/mL by MTT assay

*In-vitro drug diffusion study*

*In-vitro* drug diffusion studies were performed using Franz diffusion cell to determine the sustained release nature of the formulations. The diffusion study was continued up to 24 h, in phosphate buffer maintained (pH 7.4) at 37 ± 0.5 ºC temperature and stirred by a magnetic bar at 100 rpm under sink condition. The drug release was found to be sustained for C1, S3, D5, and O1. The percentage of cumulative drug released was of 43% for SPC: DOTAP: Tween 80 8:2:1 ratio, 41% for SPC: cholesterol: Tween 80 9:1:1 ratio and 75.4% for SPC: DOTAP: Tween 80 2:8:1 and 79.6% for SPC: DOTAP: Tween 80: cholesterol 6:2:1:3 in sustained release rate up to 24 h ( Figure 3).

*Drug release kinetics*

*In-vitro* drug release profile of the optimized formulations was then investigated by model dependent kinetics study. It was shown that Higuchi equation was the most suitable model to describe the release kinetics of lactoferrin from the transfersomal formulations. It was concluded that LF release form is square root of time dependent process. The n value is above 0.5 so that the mechanism of LF release would be suspected by usual molecular diffusion due to chemical potential grade and would be described as Fickian diffusion.

*Stability Studies*

Stability study of the prepared selected formulations showed a higher percentage of drug retained in the formulations stored at 4 °C in comparison to the samples stored at room temperature during 3 months of study. It is assumed that higher temperatures would cause higher fluidity of lipid bilayers and lead to higher drug leakage. Transferosomes also were found to be reasonably stable in terms of aggregation and fusion (Table 2).

Therefore, it can be concluded that the suitable storage condition would be cool condition of 4 °C in which the vesicle bilayers are more rigid.

*MTT assay*

MTT assay is a colorimetric assay, and it is based on conversion of yellow soluble MTT into purple insoluble formazan crystals by mitochondrial dehydrogenase. Reduction of MTT can only occur in metabolically active alive cells so that, it is a common choice in determination of cell viability and cytotoxic drug effects *in-vitro*. IC50 was obtained via measurement of Hela cell proliferation which was cultured in a 96-well plate at various BLF, T-BLF, T, and 5-FU concentrations after 72 h exposure. IC50s of BLF, T -BLF, T, and 5-FU were observed to be 5.0 mg/mL, 0.4 mg/mL, 1.95 mg/mL, and 0.6 µg/mL, respectively (Figure 4).

## Discussion

Transfersomes is a trademark which has been registered by a German based company named IDEA, AG. It means carrying bodies and it is derived from the Latin-Greek words of “transferre” and “soma” which mean “to carry across” and, “body”, respectively (26). Transfersome are categorized in two major groups including phospholipid and detergent-based types (27). An edge activator is an important part of transfersomes formulations. An edge activator is usually a single chain surfactant which destabilizes lipid bilayer and then increases the flexibility of transfersomes (28). It was claimed that transfersomes are highly deformable and can pass through the stratum corneum more easily in comparison to liposomes with one-tenth of size in some cases (27-30). They exert the hydrophilic pores and intracellular pathways with its drug cargo while retaining their integrity (26).

Transferosomes are capable of delivering low to high molecular weight drugs depending on the formulation composition, entrapment efficiency, and application conditions (27). In non-occlusive application, the deformability of the transfersomes and their non-occlusive properties allows them to follow natural water gradient across intracellular pathways of the epidermis in a sustained release and degradation protected manner (26, 31). Generally speaking, four major factors play key roles in determining the effectiveness of the elastic vesicles. These four factors are: drug interaction with the bilayers of vesicles, vesicle partitioning into the SC, the drug release from the vesicles within the SC and free drug portioning in the SC and systemic blood circulation (16, 32). Various colloid systems have been used in transdermal drug delivery for encapsulating penetrant molecules and active ingredients including liposomes, transfersomes, niosomes, ethosomes, stratum corneum lipid liposomes, cerasomes, and solid lipid nanoparticles. Following application to the skin surface, liposomes are normally reported to accumulate in the upper layers of stratum corneum with less penetration to deeper skin layers or the systemic circulation. Therefore, the classical liposomes are not useful as transdermal delivery systems but they are good choices for drug- or cosmetic-localising (33-36). On the other hand, transfersomes and ethosomes are claimed to permeate through lower skin layers to the systemic circulation. 

Liposome and transfersomes have different pharmaceutical composition. Transfersomes are composed of phospholipids, surfactants, as well as ethanol and hydration medium. Surfactants would act as a permeation enhancer by destabilizing the lipid bilayers (15, 33, 37-39). Ethanol has been shown to be an efficient skin-penetration enhancer. Ethanol interaction with the polar region of lipid bilayer would reduce the melting point of the stratum corneum lipids. This may be resulted in more fluidity and increased cell membrane permeability (15, 40). It has also been reported that formulation composition and preparation method would highly affect physiochemical characteristics and effectiveness of transfersomes in transdermal drug delivery. These include surfactant type, phospholipid types, components ratio, phospholipid: surfactant ratio, organic solvent, hydration medium, and hydration temperature. These parameters are important since they may directly influence the size, entrapment efficacy, ζ-potential, stability, and penetration properties of the prepared transfersomal vesicles (15, 39,40).

In this study we used different lipid compositions of SPC (soy phosphotidylcholine choline), cholesterol, and DOTAP. The main lipid components is unsaturated soya phosphatidylcholine (PC) with low T_m_ (transition temperatures) which become liquid crystal at high temperatures above its T_m_. Formulations with low transition temperatures would make drug leakage inevitable at room temperature or during application onto the skin. Therefore, modulation of the lipid bilayer architecture was done by optimizing ratio of lipid: surfactants and lipid compositions as well as final transition temperature (T_m_) was set between 25-32 °C for both better elasticity and prolonged storage stability. It was found that optimum ratio of SPC: DOTAP: cholesterol: tween 80 is critical in maximizing EE% and vesicle stabilities (42-48). It was seen that the higher the lipid content is, the higher EE% would be achieved. Cholesterol and DOTAP existence in lipid composition was shown to have positive effects on entrapment efficiency. It has been reported that anionic vesicles might have better permeation rate in comparison to cationic ones. Our data indicated that 1:3 ratio of DOTAP:Tween 80 would resulte in negative surface charge and higher zeta potential. Tween 80 was an anionic surfactant with HLB of 14-15 and low CMC level. Its solubilizing property and vesicle fusion prevention through increasing negative surface charge were key roles in formulation stability (46-48). This claim was confirmed by good size homogeneity and low PDI of 0.1 observed in formulated transferosomal lactoferrin (46-48). Considering drug type and its intrinsic properties such as molecular-weight (3400 dalton) and thermosensitivity, extrusion method was found to be inefficient .So the ultra sonicator probe with cold water circulation system and intermittent cycles of short pulses were selected as a complementary method for size reduction instead of extrusion (7, 45, 47).

Topically applied transfersomeal formulations interact with the skin in a sequential event plan. The difference in water content between upper and lower layers which is from 10% to 70% develops transepidermal hydration gradients. It has been stated that water affinity of the vesicle, and high elasticity are “pull” mechanism to drive vesicle toward the inner layer of the skin. But in water-rich viable epidermis, the “pull” mechanism is replaced with a “push” mechanism through diffusion-mediated mechanisms and intercellular fluid motion (7). It is noteworthy that the addition of surfactants and edge activators in transferosomal vesicle results in more deformability to penetrate across the dense SC layer even through small “pores”. In this regard, the vesicles with 100 to 1000 nm are preferred for topical/transdermal delivery. The skin permeation and skin deposition data which have not been mentioned in the current research also approved this concept. *In-vitro* efficiency was evaluated by MTT assay (49-51). The results declare that the IC50 of transfersomal lactoferrin is significantly improved in comparison to free lactoferrin which might be explained by better internalization of deformable transfersomes containing high drug content into Hela cells. 

## Conclusion

During recent years, peptides have attracted increasing interest as a new therapeutic approach. It has been reported that more than sixty peptide drugs have launched in the market, and several hundred new therapeutic peptides are now in preclinical and clinical development. However, the possible enzymatic degradation and the low cellular uptake greatly restrict the use of these compounds as pharmaceutical agents. It is clear that enhancing stability and cellular permeability are key parameters in expanding their application Transfersomes are personally designed vesicles which transverse through narrow skin pores leading to increased transdermal flux of the therapeutic agents. It is clear that transfersomes can deliver enhanced amounts of both small and large therapeutic agents into and through the skin. There are increasing applications of enhanced delivery by transfersome formulations. However, there are only two transfersome-based formulations for ketoprofen and insulin currently available in the market. But numerous applications such a delivery of anticancers, antidermititis, antimicrobial: antibacterial, antifungal, and anti-leishmaniasis, antioxidants, photo-protective agents, anti-psoriatic, anti-inflammatory and anti-acne drugs, toxoids and vaccines are now under research and development. It is likely that a number of transfersomal products for dermal and transdermal applications will be launched in pharmaceutical market in the near future.
